# Asymmetric and transient properties of reciprocal activity of antagonists during the paw-shake response in the cat

**DOI:** 10.1371/journal.pcbi.1009677

**Published:** 2021-12-28

**Authors:** Jessica R. Parker, Alexander N. Klishko, Boris I. Prilutsky, Gennady S. Cymbalyuk

**Affiliations:** 1 Neuroscience Institute, Georgia State University, Atlanta, Georgia, United States of America; 2 School of Biological Sciences, Georgia Institute of Technology, Atlanta, Georgia, United States of America; University of Pittsburgh, UNITED STATES

## Abstract

Mutually inhibitory populations of neurons, half-center oscillators (HCOs), are commonly involved in the dynamics of the central pattern generators (CPGs) driving various rhythmic movements. Previously, we developed a multifunctional, multistable symmetric HCO model which produced slow locomotor-like and fast paw-shake-like activity patterns. Here, we describe asymmetric features of paw-shake responses in a symmetric HCO model and test these predictions experimentally. We considered bursting properties of the two model half-centers during transient paw-shake-like responses to short perturbations during locomotor-like activity. We found that when a current pulse was applied during the spiking phase of one half-center, let’s call it #1, the consecutive burst durations (BDs) of that half-center increased throughout the paw-shake response, while BDs of the other half-center, let’s call it #2, only changed slightly. In contrast, the consecutive interburst intervals (IBIs) of half-center #1 changed little, while IBIs of half-center #2 increased. We demonstrated that this asymmetry between the half-centers depends on the phase of the locomotor-like rhythm at which the perturbation was applied. We suggest that the fast transient response reflects functional asymmetries of slow processes that underly the locomotor-like pattern; e.g., asymmetric levels of inactivation across the two half-centers for a slowly inactivating inward current. We compared model results with those of in-vivo paw-shake responses evoked in locomoting cats and found similar asymmetries. Electromyographic (EMG) BDs of anterior hindlimb muscles with flexor-related activity increased in consecutive paw-shake cycles, while BD of posterior muscles with extensor-related activity did not change, and vice versa for IBIs of anterior flexors and posterior extensors. We conclude that EMG activity patterns during paw-shaking are consistent with the proposed mechanism producing transient paw-shake-like bursting patterns found in our multistable HCO model. We suggest that the described asymmetry of paw-shaking responses could implicate a multifunctional CPG controlling both locomotion and paw-shaking.

## Author summary

The existence of multifunctional central pattern generators (CPGs), circuits which control more than one rhythmic motor behavior, is an intriguing hypothesis. We suggest that the cat paw-shaking response could be a transient response of the locomotor CPG. Our general prediction is that this CPG is multifunctional, and in addition to the locomotor rhythm, it can generate a transient, ten-times faster, paw-shake-like response to a stimulus. In our multistable half-center oscillator (HCO) CPG model, we applied perturbations to the locomotor pattern which resulted in a transient paw-shake-like pattern that eventually returned back to the locomotor pattern. We showed that the inactivation of the slow inward current that drives the locomotor rhythm produced asymmetry of the transient flexor and extensor activity in a symmetric HCO model. To test predictions from our model about the transient nature of the paw-shake response, we compared burst durations (BDs) and interburst intervals (IBIs) of the model half-centers in consecutive cycles of paw-shake-like responses with the BD and IBI of electromyographic (EMG) activity bursts of cat hindlimb flexors and extensors recorded during a paw-shake response. In both cases, we found similar asymmetric trends in the BD and IBI throughout a paw-shake response.

## Introduction

We engage in many types of rhythmic motor behaviors in our everyday lives, such as walking, breathing, chewing, etc. Rhythmic behaviors like these are generally controlled by interneuronal networks called central pattern generators (CPGs) [1–4]. Many of these behaviors are steady-state and long-lasting, such as walking and breathing. Other rhythmic behaviors tend to last for only a short interval of time and may have variable characteristics, such as scratching, struggling, gasping, etc. Although a wide variety of motor rhythms have been described in the literature, the mechanisms underlying these rhythms are still debated. For example, the relatively slow locomotor rhythm and the much faster paw-shake response in the cat could be controlled by two separate independent CPGs, separate CPGs with shared interneuronal circuits, or a single multifunctional CPG. There is experimental evidence from a variety of species supporting the possibility of multifunctional CPGs controlling a variety of different motor behaviors [5–13].

In principle, multifunctional CPGs could produce multiple rhythms of different frequencies. Relatively slow long-lasting rhythmic behaviors could be controlled by a stable, steady-state rhythm of a multifunctional CPG until an input signal changes or halts its rhythmic activity. A short-duration, non-steady-state rhythmic behavior, on the other hand, could result from transient neural dynamics of the same CPG triggered by a perturbation, e.g., a sensory input signal. These transient neural dynamics could generate a short-duration rhythmic behavior as a reliable response to certain environmental stimuli, such as transient responses seen in insect olfaction processing [14–16].

We have recently developed a multifunctional CPG model that generates reciprocal rhythmic activity between cat hindlimb antagonists and exhibits multistability of slow, locomotor-like and fast, paw-shake-like steady-state rhythms [17,18]. The model is based on a novel mechanism that produces multistability of two rhythms that differ in frequency by an order of magnitude, e.g., 1 Hz and 10 Hz. This mechanism involves two slow, intrinsic currents in each half-center neuron with significantly different kinetics of inactivation. These two currents are slowly inactivating, low-voltage-activated calcium current (*I*_*CaS*_) and slowly inactivating sodium current (*I*_*NaS*_). The locomotor-like rhythm in our model is largely generated and controlled by *I*_*CaS*_, while the paw-shake-like rhythm is largely generated and controlled by *I*_*NaS*_. Although *I*_*CaS*_ and *I*_*NaS*_ both inactivate relatively slowly, the time constant of inactivation of *I*_*CaS*_ is much larger than the time constant of inactivation of *I*_*NaS*_. The slow calcium current in our model deinactivates at voltages that are more hyperpolarized than the minimum voltage reached during the stable paw-shake-like rhythm. Therefore, *I*_*CaS*_ is completely inactivated during the paw-shake-like rhythm, allowing *I*_*NaS*_ to control the paw-shake-like rhythm and maintain a much higher frequency than the locomotor-like rhythm. During the locomotor-like rhythm, however, *I*_*CaS*_ and *I*_*NaS*_ are both active, but the kinetics of *I*_*CaS*_ bring the rhythm to a much slower frequency. With a short perturbation (a pulse of conductance) to one or both half-center neurons, the model can be switched from the locomotor-like rhythm to the paw-shake-like rhythm or vice versa. We have shown that a neuromechanical model of cat hindlimbs controlled by this multifunctional CPG and motion-dependent somatosensory feedback could reproduce not only distinct rhythms of cat locomotion and paw-shaking but also the typical reciprocal activation of flexors and extensors during locomotion and the atypical reciprocal activation of anterior and posterior muscles during paw-shaking [17,19]. In a previous study, we modeled steady-state paw-shake-like activity patterns [18], whereas recorded paw-shake cycle periods are not steady but become longer with each subsequent paw-shake cycle [19]. This suggests that paw-shake responses could be generated by transient dynamics elicited by some sensory stimuli. Here, we investigated potential mechanisms that can produce transient paw-shake-like responses to short pulses of current applied at various phases of locomotor-like activity of our previously developed multistable, half-center oscillator CPG model [17,18]. We also compared characteristics of the model’s transient dynamics with the characteristics of EMG activity recorded during paw-shake responses in unrestrained cats.

Thus, the first goal of this study was to investigate the mechanism by which neural transient dynamics could be produced in the multistable, half-center oscillator CPG model. The second goal was to investigate temporal characteristics of EMG activity of cat hindlimb antagonistic muscles (durations of activity cycles, bursts and interburst intervals) in consecutive paw-shake cycles evoked during locomotion and compare them with those of the model. We tested the hypothesis that the temporal characteristics of recorded EMG activities during cat paw-shake responses would be consistent with the model transient dynamics.

## Methods

### Ethics statement

All surgical and experimental procedures of this study were in compliance with the “Guide for the Care and Use of Laboratory Animals. Eighth Edition” (National Research Council, 2011) and were approved by the Institutional Animal Care and Use Committee of the Georgia Institute of Technology (protocol number A13063).

### Modeling

We used our previously published Hodgkin-Huxley style model [17,18] to investigate fast paw-shake-like activity as a transient response to a perturbation of a steady-state locomotor-like rhythm. Our model consists of two identical, mutually inhibiting neurons with the same intrinsic currents and the same synaptic currents. There are five intrinsic currents in each neuron: a fast sodium current (*I*_*NaF*_), a slowly inactivating sodium current (*I*_*NaS*_), a potassium current (*I*_*K*_), a low-voltage-deinactivated, slowly inactivating calcium current (*I*_*CaS*_), and a leak current (*I*_*leak*_). Currents *I*_*NaF*_ and *I*_*K*_ were modified from another mammalian locomotion CPG model [20,21]. The following equations use the units of second (s), milliVolt (mV), nanoSiemens (nS), and picoAmpere (pA):

$$V^{\prime }=-\frac{1}{C}[I_{NaF}+I_{NaS}+I_{K}+I_{CaS}+I_{leak}+I_{Syn}+I_{E}]$$

$${I_{NaF}=\bar{g}}_{NaF}m_{NaF,\infty }^{3}h_{NaF}[V-E_{Na}]$$

$${I_{NaS}=\bar{g}}_{NaS}m_{NaS}h_{NaS}[V-E_{Na}]$$

$${I_{K}=\bar{g}}_{K}{m_{K}}^{4}[V-E_{K}]$$

$${I_{CaS}=\bar{g}}_{CaS}m_{CaS}^{3}h_{CaS}[V-E_{Ca}]$$

$${I_{leak}=g}_{leak}[V-E_{leak}]$$

$${I_{Syn}=\bar{g}}_{Syn}m_{SynPre}[V-E_{Syn}]$$

$$m_{NaF,\infty }=\frac{1}{1+exp(\frac{V+20}{-7.8})}$$

$$I_{E}=g_{E}(t)[V-E_{E}],\;g_{E}(t)=\left\{ \begin{array}{c} {\bar{g}}_{E},\;t_{pul}\leq t\leq t_{pul}+d_{pul}\\ 0,\;t<t_{pul}\;or\;t>t_{pul}+d_{pul} \end{array}\right.$$

$${h^{\prime }}_{NaF}=\left[\frac{1}{1+exp(\frac{V+23}{7})}-h_{NaF}\right]/\left[\frac{.03}{exp(\frac{V+40}{15})+exp(\frac{V+40}{-16})}\right]$$

$${m^{\prime }}_{NaS}=\left[\frac{1}{1+exp(\frac{V+42}{-4.1})}-m_{NaS}\right]/.001$$

$${h^{\prime }}_{NaS}=\left[\frac{1}{1+exp(\frac{V+55}{5})}-h_{NaS}\right]/.1$$

$${m^{\prime }}_{K}=\left[\frac{1}{1+exp(\frac{V+21}{-15})}-m_{K}\right]/\left[\frac{.007}{exp(\frac{V+46}{40})+exp(\frac{V+46}{-50})}\right]$$

$${m\prime }_{CaS}=\left[\frac{1}{1+exp(\frac{V+45.59}{-4.27})}-m_{CaS}\right]/\left[\frac{.001}{\left[\frac{.02*[V+48]}{1-exp(\frac{V+48}{-4.5})}]+[\frac{-.05*[V+51]}{1-exp(\frac{V+51}{4.5})}\right]}\right]$$

$${h^{\prime }}_{CaS}=\left[\frac{1}{1+exp(\frac{V+58.93}{.75})}-h_{CaS}\right]/.485$$

$${m^{\prime }}_{SynPre}=\left[\frac{1}{1+exp(\frac{V_{Pre}}{-0.4})}-m_{SynPre}\right]/.009,$$

where ${\bar{g}}_{x}$ is the maximal conductance; *E*_*x*_ is the reversal potential; *m*_*x*_ and *h*_*x*_ are the activation and inactivation variables, respectively, of some current *I*_*x*_. The activation and inactivation variables range from 0 to 1, where the current is fully deactivated when *m*_*x*_ = 0 and fully activated when *m*_*x*_ = 1; the current is fully inactivated when *h*_*x*_ = 0 and is fully deinactivated when *h*_*x*_ = 1 [22]. The variable *m*_*SynPre*_ is the synaptic activation variable of the presynaptic neuron which is governed by the corresponding presynaptic membrane potential *V*_*Pre*_. The capacitance *C* in this model is 0.001 nF.

We integrated these equations using the 8th order Runge-Kutta integration method and GSL ODE solver software, with a maximum step size of 10^−5^ seconds, an absolute tolerance of 10^−8^ and relative tolerance of 10^−9^. Values of the model parameters are provided in Tables 1 and 2.

**Table 1:** Canonical conductance parameters and perturbation conductance (in nS).

${\bar{g}}_{NaF}$	${\bar{g}}_{NaS}$	${\bar{g}}_{K}$	${\bar{g}}_{CaS}$	*g*_*leak*_	${\bar{g}}_{Syn}$	${\bar{g}}_{E}$
50.0	3.83	40.0	12.3	2.96	5.5	1.0

**Table 2:** Reversal potential parameters and perturbation reversal potential (in mV).

*E*_*Na*_	*E*_*K*_	*E*_*Ca*_	*E*_*leak*_	*E*_*Syn*_	*E*_*E*_
65.0	-70.0	160.0	-54.0	-75.0	0.0

The transient fast rhythmic activity was elicited in our model by applying a pulse of an additional excitatory modulatory current, *I*_*E*_ with an excitatory reversal potential *E*_*E*_ = 0 mV, as a square pulse of conductance, *g*_*E*_. This pulse of modulatory current was applied to both neurons in the model when the model was engaged in a slow locomotor-like rhythm. It was applied at a specified time, *t*_*pul*_, corresponding to a specific phase, *p*_*pul*_, and it continued for a specified duration, *d*_*pul*_. The fast rhythmic activity that results from this pulse may be either transient or stable. Many cases of transient paw-shake-like activity were collected by varying the time of pulse onset, *t*_*pul*_, and the pulse duration, *d*_*pul*_. The phase *p*_*pul*_ = 0% was defined as the instant of the first spike peak in each burst of neuron 2. We varied *p*_*pul*_ from 0% to 100% with a step of 0.25% of the cycle, and for each of these phases, we varied pulse duration *d*_*pul*_ from 200 ms to 1200 ms with a step of 5 ms.

For each episode of transient paw-shake-like activity in the model, we measured the temporal characteristics of bursting activities of the two neurons comprising the half-center oscillator CPG for each subsequent activity cycle. These characteristics included bursting cycle period (CP), burst duration (BD), and interburst interval duration (IBI). CP of each neuron was calculated as the time between the peak of the first spike of a burst and the peak of the first spike of the next burst. BD was calculated as the time between the peak of the first spike of a burst and the peak of the last spike of the same burst. IBI was defined as (CP–BD). Duty cycle (DC) was defined as 100*BD/CP. Paw-shake-like rhythms were defined as rhythms with CPs < 210 ms. The progressions of BD and IBI throughout each episode of transient activity were measured for each neuron and each phase of pulse onset. Linear regression analysis was used to perform a linear curve fit in order to evaluate the rate of change of BD and IBI over time during transient activity. We excluded the very first burst after the pulse from the linear curve fit, because there were some simulations where the first burst was very short, sometimes consisting of only 2 or 3 short spikes. For each 0.25%-phase increment of pulse onset, we obtained a simulation of transient paw-shake-like activity. Among all collected responses, we selected and used for further analyses those which contained between 5 and 9 paw-shake-like burst cycles, and disregarded simulations with fewer or greater. We applied linear curve fits to BD and IBI versus time for each collected simulation, determined the slopes of these fits for each simulation, and used a sliding averaging of the slopes with a window width of 1.25% of the cycle duration. That gave us the mean slope values of BD and IBI for all simulations corresponding to each perturbation phase. We also investigated more closely the mean slopes of BD and IBI in the phase range of 20% - 40% which approximately corresponds to the locomotor cycle phases in which paw-shaking is actually initiated in cats.

We hypothesized that the asymmetry of the linear trends of BD and IBI throughout time in our model could be explained by the functional antiphase relationships between the slowest state variables of the two half-centers. To test this hypothesis, we built a reduced version of the model, which we called the “constant *h*_*CaS*_ model”. In each neuron of the reduced model, the *h*_*CaS*_ state variable was held constant and its corresponding equations were removed from the CPG model. We separately varied *h*_*CaS1*_ and *h*_*CaS2*_ as two parameters, producing a simulation of the model for each combination of values in a certain domain. Each simulation was integrated for 1000 s to ensure that the activity had reached a steady state. We obtained the mean values of BD and IBI for neurons #1 and #2 separately by averaging over the last 20 bursts for each simulation case. We measured the difference of DC between the two model neurons and plotted this difference versus the difference in *h*_*CaS*_ of the two neurons, and we performed a linear curve fit on these data.

### Animal experiments

We analyzed EMG recordings from 12 adult female cats (mass 3.56±0.73 kg) that were part of our previous studies [23–26]. Details of surgical and experimental procedures were described previously [23,24]. Briefly, cats were trained with food rewards to walk on a plexiglass enclosed walkway. After training, the major ankle, knee and hip muscles of one hindlimb were implanted with bipolar wire electrodes (CW5402; Cooner Wire, Chatsworth, CA, USA) under aseptic conditions and general isoflurane anesthesia (1–3%). The animals recovered after surgery for 2 weeks. Pain medication (fentanyl transdermal patch, 12–25 μg/h and/or buprenorphine, s.c., 0.01 mg/kg, or ketoprofen, 2 mg/kg, s.c.) and antibiotics (cefovecin, 8 mg/kg, s.c., or ceftiofur, 4 mg/kg, s.c.) were administered for 3 and 10 days, respectively. To evoke paw-shake responses during walking, we attached a small piece of adhesive tape (~2 cm x 3 cm) to paw pads of the implanted hindlimb and placed the cat on the walkway. The animal walked across the walkway and performed paw-shaking during the swing phase of the hindlimb keeping the other limbs on the ground until termination of paw-shaking and resuming locomotion [23,27]. EMG activity was recorded at a sampling rate of 3000 Hz. Recorded EMG signals were band pass filtered (30–1000 Hz) and amplified. Since our CPG model describes the operation of a HCO that acts as a rhythm generator and generates excitatory inputs to the neural network that activates flexor and extensor motoneurons, we analyzed paw-shake EMG recordings from hindlimb muscles with flexor and extensor locomotor activity (25). These muscles included flexors *tibialis anterior* (TA, ankle flexor) and *iliopsoas* (IP, hip flexor) and extensors *soleus* (SO, ankle extensor), *medial gastrocnemius* (MG, ankle extensor and knee flexor), *vastus lateralis* and *vastus medialis* (VA, knee extensor), and *anterior biceps femoris* (BFA, hip extensor).

For each of these muscles, we measured the progression of EMG burst characteristics of paw-shake cycles throughout each paw-shake episode. In order to measure these burst characteristics, we first full-wave rectified the EMG signal and slightly smoothed it using a zero-lag, moving average with an 8-ms window. For each channel, we then took a random time interval with no muscle activity that was at least 200 ms wide and determined the mean and standard deviation of the raw EMG signal within this selection. The standard deviation was used as a measure of the EMG noise level in each trial. We set the activity burst threshold to be 3 times this standard deviation. We defined the EMG paw-shake cycle period (CP) for each muscle as the time between the EMG burst onset in a given cycle and the burst onset in the next cycle. The EMG burst duration BD was defined as the time between the burst onset and offset, and EMG interburst interval IBI was defined as the time between the burst offset and next burst onset. The duty cycle DC was calculated as 100*BD/CP.

We analyzed changes in BD and IBI in all consecutive cycles of individual paw-shake episodes for each muscle: 28 episodes for MG, 28 for SO, 12 for VA, 12 for BFA, 21 for IP, and 12 for TA (Table 3). Each paw-shake episode had between 5 and 11 cycles. We calculated Pearson’s correlation coefficient for BD and IBI versus time for each paw-shake response within each muscle group. The median Pearson’s correlation coefficient (R) of all trials within each muscle group was greater than 0.7 for IBI of MG, SO, VA, and BFA; and for BD of IP, TA, and VA. For the IBI of IP and TA and for BD of MG, SO and BFA, BD/IBI appeared to remain constant over time, and therefore, R varied widely from trial to trial with a median R anywhere from -0.3 to 0.3. We used linear regression analysis of BD and IBI versus time for each paw-shake response to see if the median curve fit slopes were statistically different from zero in the case of variables that appeared to remain constant and to quantify the mean and median rates of change of these variables over time in the case of groups with high R values. In order to be consistent with the model analysis, we excluded the first burst from the regression analysis. We then calculated the slopes of these regression lines for each muscle and paw-shake episode. We determined that we could not assume the regression slopes were normally distributed based on histograms of the slope data and eyeball metrics. We used the one-sample non-parametric Wilcoxon rank test to determine whether the median linear regression slopes were significantly different from zero.

**Table 3:** Number of paw-shake episodes analyzed per animal and muscle.

Animal ID	MG	SO	VA	BFA	IP	TA
A	8	7	2	0	5	0
B	7	6	0	0	2	0
C	6	7	6	9	6	0
D	2	2	0	2	0	0
E	1	2	0	0	0	0
F	1	0	0	0	2	6
G	1	1	0	0	1	0
H	1	0	0	0	0	0
I	1	0	0	0	0	0
J	0	2	2	1	3	0
K	0	1	0	0	1	2
L	0	0	2	0	1	4
Total	28	28	12	12	21	12

Muscle abbreviations: MG, medial gastrocnemius; SO, soleus; BFS, biceps femoris anterior; IP, iliopsoas; TA, tibialis anterior; VA, vastus lateralis and vastus medialis. Each paw-shake episode contained between 5 and 11 cycles. The raw data could be found in S1 Data.’

## Results

### Transient dynamics of half-center oscillator model

**Fig 1:**
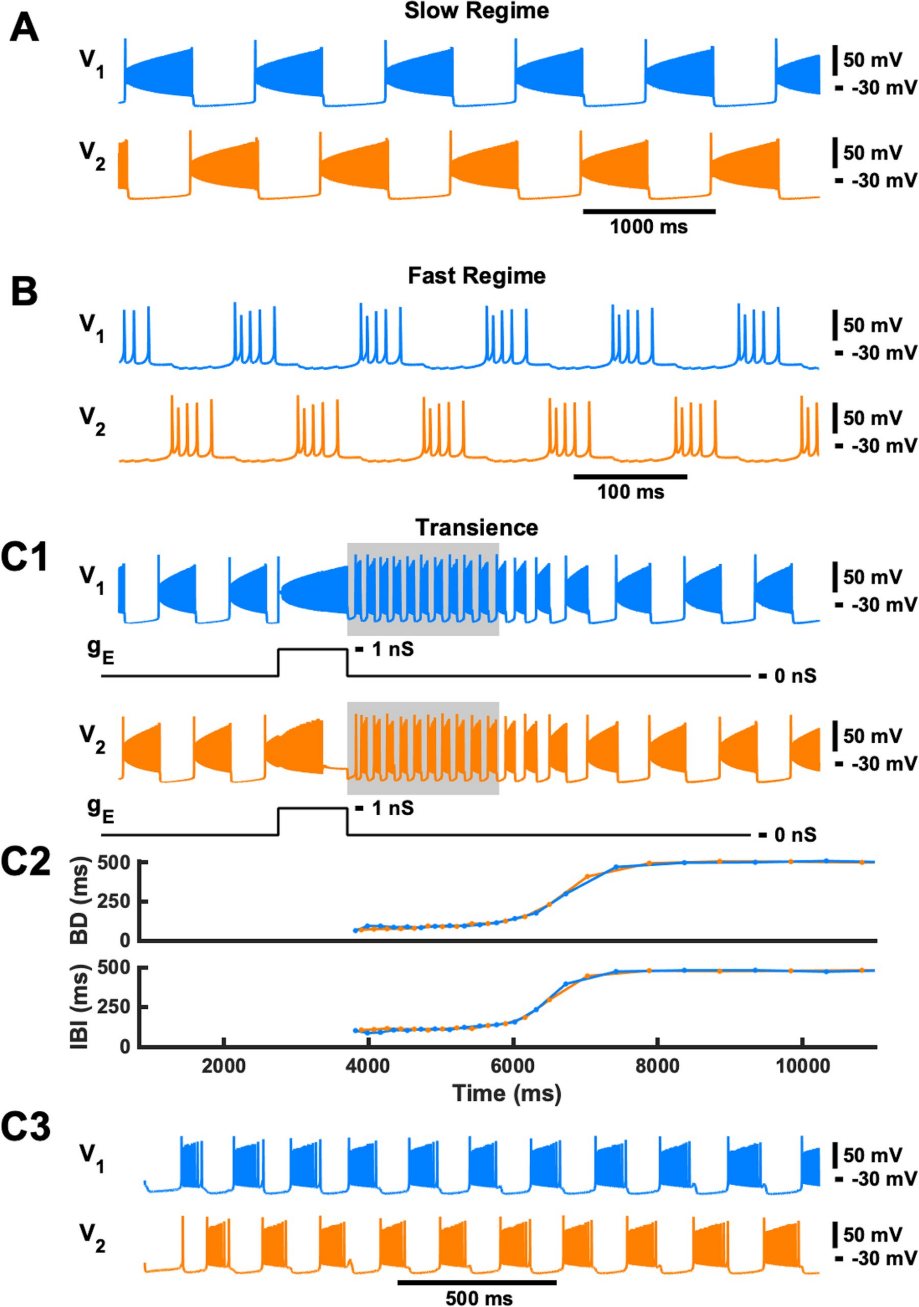
Examples of stable and transient activity in a symmetric HCO model. Characteristics of neuron #1 are depicted in blue, while characteristics of neuron #2 are depicted in orange. **A and B**: A slow locomotor-like rhythm **(A)** and a fast paw-shake-like rhythm **(B)** coexist in the model. **(C1)** A transient fast rhythm was elicited by applying a square pulse of excitatory conductance with amplitude ${\bar{g}}_{E}$ = 1 nS to both neurons for about 1000 ms (950 ms was used in this example). The pulse was applied during the spiking phase of neuron 2 with the phase of pulse onset equal to 20%. The transient fast rhythmic activity occurs immediately after the pulse and activity returns to the slow rhythm in about 2 seconds after the pulse offset. The gray rectangle represents the region which is depicted in **C3**. **(C2)** The BD and IBI of neurons #1 are #2 during the transient response. The time axis is common for **C1 and C2**. **(C3)** Zooming in on the transient paw-shake-like activity marked by the gray rectangle in **C1**.

**Fig 2:**
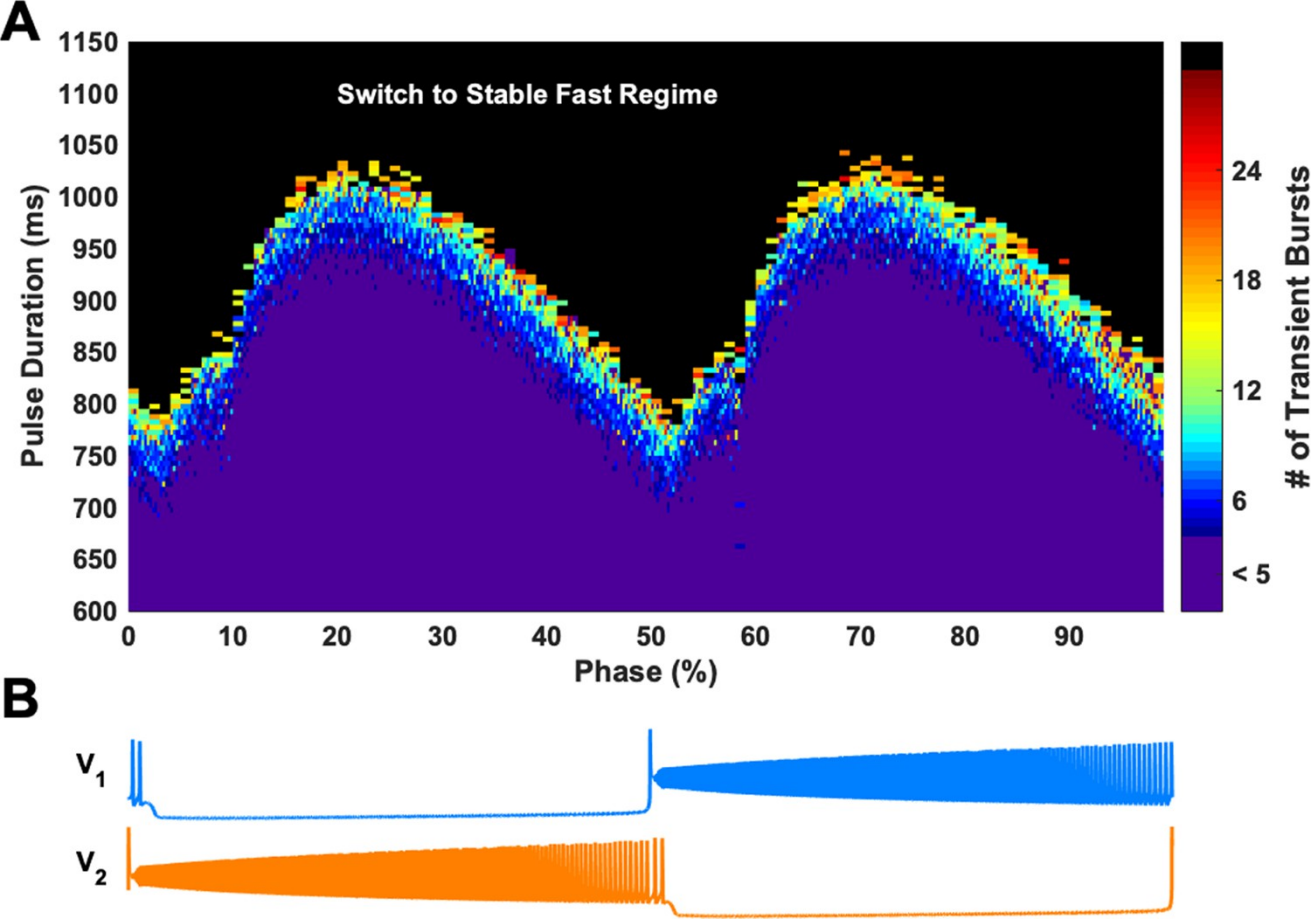
The number of bursts contained in a transient response depends on the phase of pulse onset and pulse duration. The color map represents the duration of transient fast rhythm response (measured as the number of transient bursts). The black region above the colored portion represents traces where the pulse caused a switch to the stable fast rhythm. The locomotor-like cycle corresponding to the phase of pulse onset during the slow rhythm is shown at the bottom by one full bursting cycle period of neurons #1 (blue trace) and #2 (orange trace).

In our model of a half-center oscillator, two steady-state oscillatory regimes–slow locomotor-like and fast paw-shake-like, coexist (Fig 1A and 1B), as previously described in detail in [17,18]. In previous studies, we simulated a paw-shake response during locomotion by applying a pulse of current to switch from stable locomotor-like activity to stable paw-shake-like activity and then applying another pulse of current to switch back to locomotor-like activity. Here, we reduced the duration of the first pulse in order to produce a transient paw-shake-like response, where a second pulse is not needed to return to locomotor-like activity (Fig 1C). In this case, the model produced a few paw-shake-like bursts and then slowly (within several seconds) returned to a slow steady-state locomotor-like activity. During this transient activity, BD and IBI of both neurons changed as a sigmoid function of time (Fig 1C). The duration of the transient paw-shake-like activity (i.e., the number of transient bursts) depended on the phase of pulse onset and the pulse duration (Fig 2). The pulse duration needed to produce this transient activity also changes depending on the phase of pulse onset (Fig 2).

**Fig 3:**
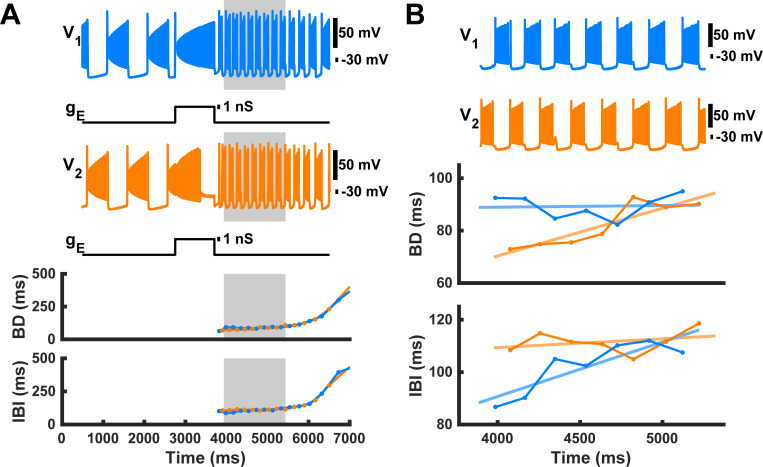
Example of burst duration (BD) and interburst interval duration (IBI) progressions throughout transient paw-shake-like activity. The membrane potential, BD, and IBI of neurons #1 and #2 are depicted in blue and orange, respectively. **(A)** Revisiting the example from Fig 1, where the gray region indicates transient paw-shake-like activity. **(B)** Zooming in on the gray region from panel **A**, we see that BD_2_ (orange lines) and IBI_1_ (blue lines) increase, while BD_1_ (blue lines) and IBI_2_ (orange lines) remain roughly constant.

In large regions of phase space, which will be explored further in the following, we found asymmetric trends across the two half-centers within transient paw-shake-like activity, despite that our HCO CPG model had two completely symmetric half-centers (two neurons with the same intrinsic and synaptic properties) with identical burst characteristics for the steady-state slow regime and the fast regimes and that both neurons received the same perturbations at the same time. Looking back at the example simulation in Fig 1, when we zoomed in on transient paw-shake-like activity, we found that the burst characteristics of the two neurons evolved asymmetrically throughout the transient paw-shake-like activity (Fig 3). If the pulse was applied during the spiking phase of the locomotor-like burst of neuron #2, then its BD (BD_2_) grew faster than the BD of neuron #1 (BD_1_), and the IBI of neuron #1 (IBI_1_) grew faster than the IBI of neuron #2 (IBI_2_) (Fig 3B). For each simulation of transient paw-shake-like activity, we computed linear regressions between BD and the time of burst occurrence within the paw-shake-like response, i.e. the burst onset time, between IBI and the burst onset time, and between DC and burst onset time.

**Fig 4:**
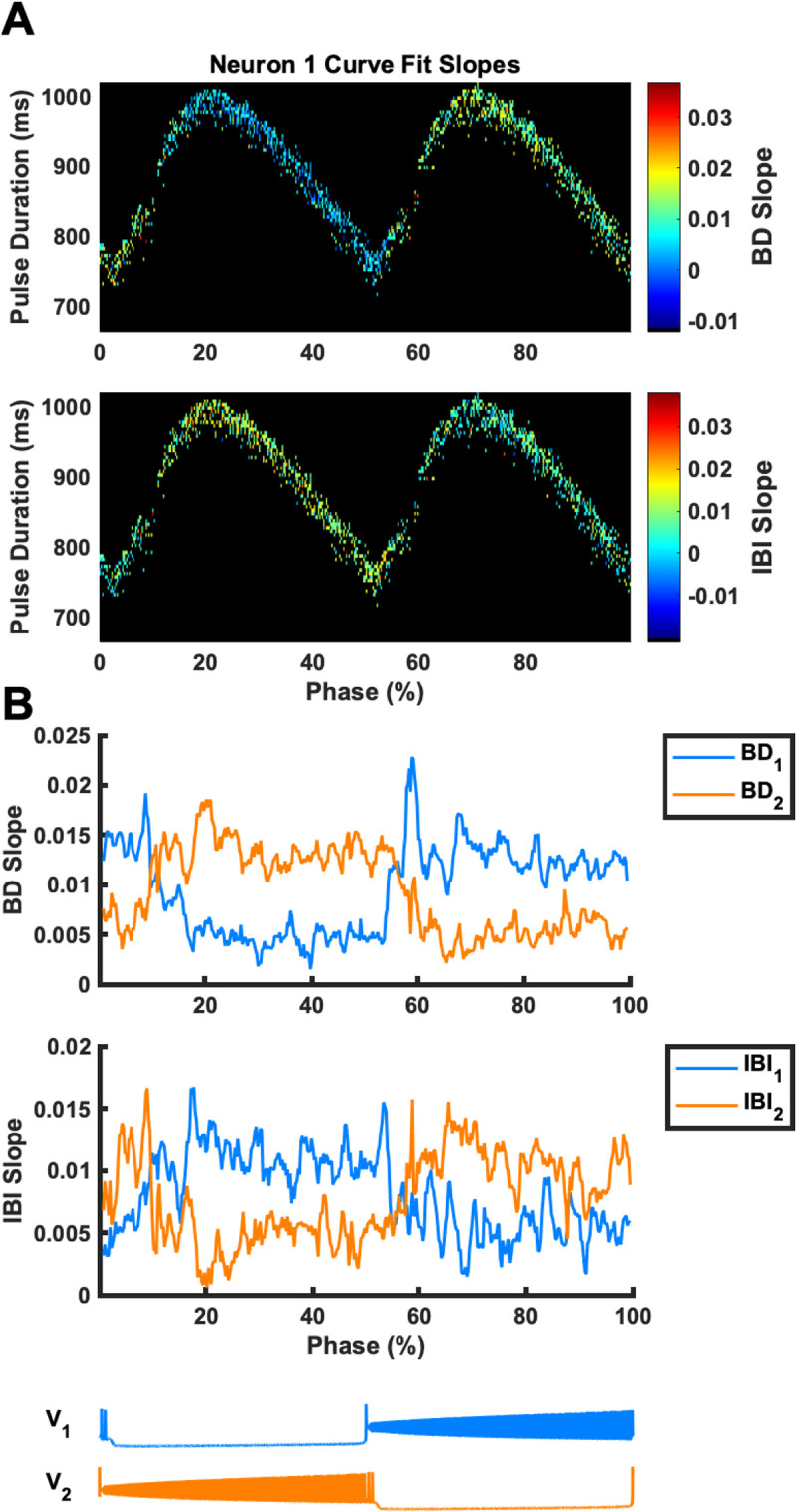
Regression slopes of the relationship between burst duration and burst onset time (BD slope) and between interburst interval and burst onset time (IBI slope) throughout paw-shake-like episodes depend on phase of pulse onset and pulse duration. **(A)** BD slope and IBI slope for neuron #1 are shown for simulations with 5 to 9 transient paw-shake-like bursts. The color map represents the magnitude of these slopes. **(B)** BD slope and IBI slope averaged across pulse durations as a function of phase of pulse onset for neuron #1 and neuron #2 and smoothed with a sliding window of phase width 1.25%. A locomotor-like cycle corresponding to the phase of pulse onset is shown at the bottom by traces of neurons #1 and #2.

We then collected all simulations from the analysis in Fig 3 that had between 5 and 9 paw-shake cycles, which amounted to 1328 model simulations relatively evenly distributed across phase of pulse onset (Fig 4A). In this data set, we found that the regression slopes (the mean rate of change) of BD and IBI for neurons #1 and #2 depended on the phase of pulse onset and pulse duration (Fig 4A). There was substantial variability in the slopes for BD and IBI as evident from Fig 4B, so we used a sliding window method to produce a relatively smoothed, averaged representation of the slopes of BD and IBI versus phase of pulse onset disregarding pulse duration (Fig 4B). At around phase = 10% (near the burst onset of neuron #2), BD_2_ slope became greater than BD_1_ slope, and at around phase = 55% (near the burst onset of neuron #1), BD_1_ slope became greater than BD_2_ slope. On the other hand, at around phase = 15%, IBI_1_ slope became greater than IBI_2_ slope, and at around phase = 60%, IBI_2_ slope became greater than IBI_1_ slope.

### Asymmetric responses of the symmetric HCO model

**Fig 5:**
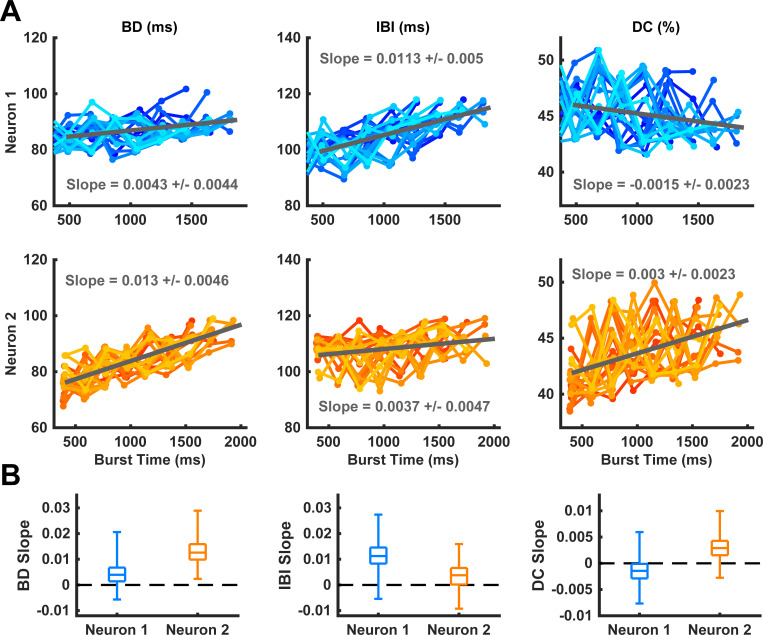
Simulated burst durations (BD), interburst intervals (IBI), and duty cycles (DC) and their linear regression lines as functions of the burst onset time during transient paw-shake-like responses. We generated 286 different cases of transient activity by applying a pulse of conductance with different pulse durations (between 700 ms and 1000 ms) and at different phases of locomotor-like activity of neuron #2 (between 20% and 40%). **(A)** Only 20 randomly chosen episodes of the paw-shake-like activity from these simulations are shown here to avoid congestion; the shown regression lines were computed using all 286 episodes. For each simulation, a linear regression line was computed for BD, IBI, and DC of neurons #1 and #2, and the average slope of these regression lines was calculated (shown in top and middle rows for neurons #1 and #2, respectively). **(B)** The median and quartile slopes are shown for BD slope, IBI slope, and DC slope.

We then looked more closely at a range of pulse application phases from 20% to 40% where behaviors of BD and IBI in the model and cat experiments were similar (see next section) and where actual paw-shake responses during cat locomotion begin, i.e. in the flexor (swing) phase of a locomotion cycle assuming neurons #1 and #2 represent the extensor and flexor half-centers, respectively. Within this range, the pulse was applied around the middle of the spiking activity of neuron #2, which would represent the swing phase of a cat’s locomotion cycle. We produced 286 simulations in this phase range by modifying the phase of pulse onset and the pulse duration, so that the pulse duration ranged from 700 ms to 1000 ms. Slightly varying these pulse parameters produced highly variable effects on the resulting transient activity of neurons #1 and #2 (Figs 4 and 5). We calculated the mean and median slopes of the regression lines for all of the simulations in this range and found that the median slopes of the linear regression lines for BD, IBI and DC as functions of the burst onset time during paw-shake-like activity were significantly different from zero for neuron #1 and neuron #2. However, the median BD slope of neuron #1 (0.0043) was significantly smaller than the median BD slope of neuron #2 (0.013). The median IBI slope of neuron #2 (0.0037) was significantly smaller than the median IBI slope of neuron #1 (0.011). Moreover, the DC slopes for neurons #1 (-0.0015) was negative, while the DC slope for neuron #2 was positive (0.003) (Fig 5). Thus, despite the identical properties of the two half-centers of the HCO model, they produced asymmetric transients in response to identical perturbation pulses.

**Fig 6:**
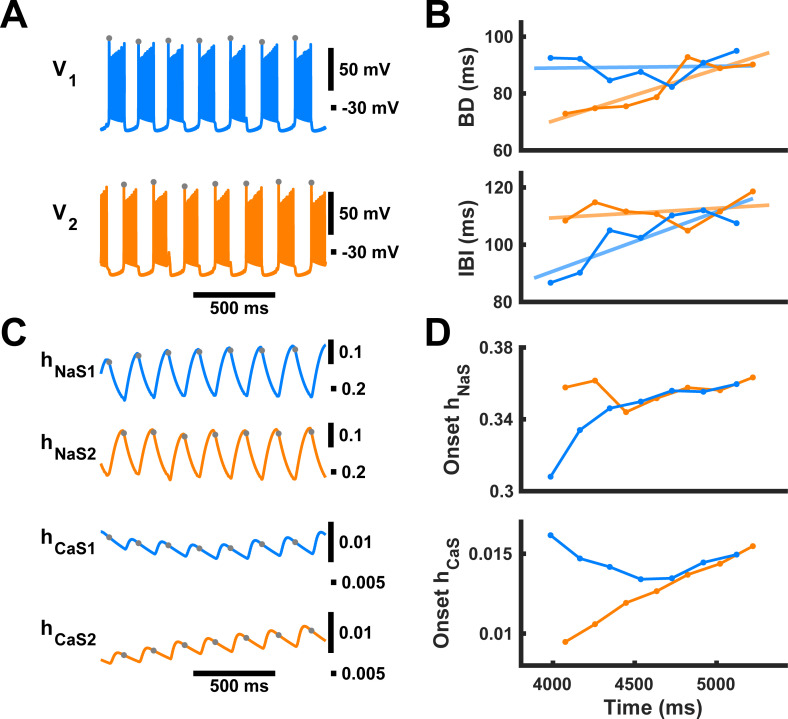
Progression of variables *h*_*NaS*_ and *h*_*CaS*_ throughout transient paw-shake-like response. Characteristics for neurons #1 and #2 are depicted in blue and orange, respectively. All panels are on the same time scale. **(A)** Membrane potentials during model transient paw-shake-like activity from the same simulation shown in Fig 3. Gray dots represent the time of the burst onset. **(B)** Changes in burst duration (BD) and interburst interval (IBI) of neurons #1 and #2 in an example of a paw-shake-like transient response, also shown in Fig 3B. **(C)** Progression of variables *h*_*NaS*_ and *h*_*CaS*_ during transient-paw-shake-like activity. **(D)** The values of variables *h*_*NaS*_ and *h*_*CaS*_ for neurons #1 and #2 at the burst onsets of each neuron, which we refer to as “onset *h*_*NaS*_” and “onset *h*_*CaS*_”, marked by gray dots in **A** and **C**.

We then investigated possible causes of the asymmetric changes of BD and IBI in our symmetric half-center oscillator CPG model. We looked at the differences in inactivation of the two slow currents, *h*_*NaS*_ (time constant of 100 ms) and *h*_*CaS*_ (time constant of 485 ms) between the two neurons throughout the transient paw-shake-like response (Fig 6). Although values of *h*_*NaS*_ at the onset of each burst were asymmetric for the first couple bursts, they did not exhibit substantial asymmetry throughout the entire duration of transient activity and thus could not explain the differences in temporal evolution of BD and IBI between the two neurons (Fig 6B and 6D). On the other hand, the values of *h*_*CaS*_ of neurons #1 and #2 progressed differently throughout the entire duration of transience (Fig 6C). Although the difference in the *h*_*CaS*_ values at burst onset between the two neurons was small (Fig 6D), we hypothesized that it could explain the asymmetric evolution of BDs and IBIs throughout the transient activity. While *h*_*NaS*_ may play some role in the initial asymmetry in BD and IBI across the two neurons, mainly at the beginning of transient activity, we focused on the role of *h*_*CaS*_ due to the clear asymmetry in the progression of *h*_*CaS*_ throughout the entire duration of transient-paw-shake-like activity (Fig 6C and 6D).

**Fig 7:**
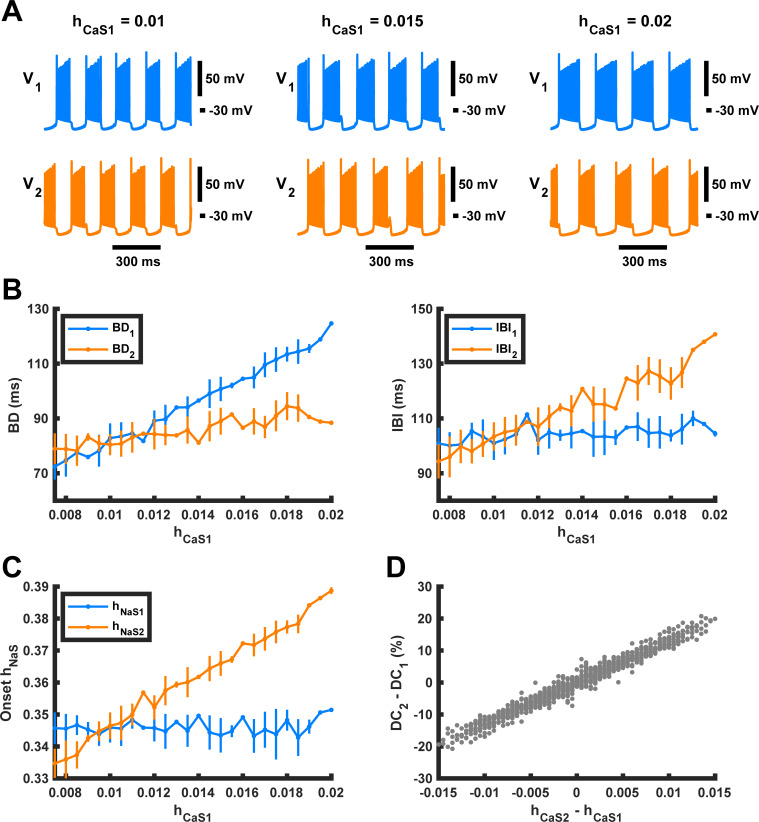
Dependence of burst duration (BD) and interburst interval (IBI) in model HCO neurons #1 and #2 on *h*_*CaS1*_. **(A)** Bursting paw-shake-like activity in neurons #1 (blue trace) and #2 (orange trace) at different values of parameter *h*_*CaS1*_, while the value of parameter *h*_*CaS2*_ remained constant at 0.01. **(B)** In the reduced constant-*h*_*CaS*_ model, BD_1_ and IBI_2_ increased substantially with increasing *h*_*CaS1*_, while IBI_1_ and BD_2_ only slightly increased. (C) The onset *h_NaS2_* increased linearly while onset *h_NaS1_* stayed roughly constant. **(D)** The difference in duty cycle between the two neurons depends linearly on the difference in *h*_*CaS*_ between the two neurons. This dependence was calculated using every simulated combination of *h*_*CaS1*_ and *h*_*CaS2*_, where both *h*_*CaS*_ parameters were varied from 0.0075 to 0.02 with steps of 0.0005.

To further investigate the effects of the difference in *h*_*CaS*_ between two neurons, we built a reduced version of the model, called the “constant *h*_*CaS*_ model”, in which *h*_*CaS*_ for each neuron was treated as a parameter, and the corresponding variable and its differential equation was removed from the neuron model. These new parameters, *h*_*CaS1*_ and *h*_*CaS2*_, corresponding to the inactivation of slow calcium current in neurons #1 and #2, respectively, were kept constant with respect to time. We varied *h*_*CaS1*_ and *h*_*CaS2*_ separately, to produce a simulation of the model for each combination of values. We first kept *h*_*CaS2*_ constant at 0.01, and varied *h*_*CaS1*_ from 0.0075 to 0.02 by steps of 0.0005. We found that BD of neuron #1 increased with the parameter value of *h*_*CaS1*_, while IBI of neuron #1 increased only slightly as *h*_*CaS1*_ increased (Fig 7A and 7B). On the other hand, BD of neuron #2 only slightly increased as *h*_*CaS1*_ increased, while IBI of neuron #2 increased significantly as *h*_*CaS1*_ increased (Fig 7A and 7B). We measured mean onset *h*_*NaS*_ for both neurons as we varied *h*_*CaS1*_ and found that as *h*_*CaS1*_ increased, onset *h*_*NaS2*_ increased linearly while onset *h*_*NaS1*_ remained roughly constant, reflecting changes in corresponding constant values of parameters *h*_*CaS1*_ and *h*_*CaS2*_ (Fig 7C).

Then for each value of *h*_*CaS1*_, we varied *h*_*CaS2*_ between 0.0075 and 0.02 with a step size of 0.0005, and we used the same range of values and step size to vary *h*_*CaS1*_. We found that within this data set of 676 simulations the difference in duty cycle between the two neurons depended linearly on the difference in *h*_*CaS*_ between the two neurons in the reduced model (Fig 7D). This means that the difference in BD between the two neurons normalized by their shared cycle period depends linearly on the difference in *h*_*CaS*_ of the two neurons. Therefore, the duty cycles are directly and predictably controlled by the difference in *h*_*CaS*_ (*h*_*CaS2*_*-h*_*CaS1*_) between the two neurons. We concluded that the asymmetry in transient responses in the full model occurred due to the difference in how much the slow calcium currents in both neurons inactivate while they are depolarized during the pulse.

**Fig 8:**
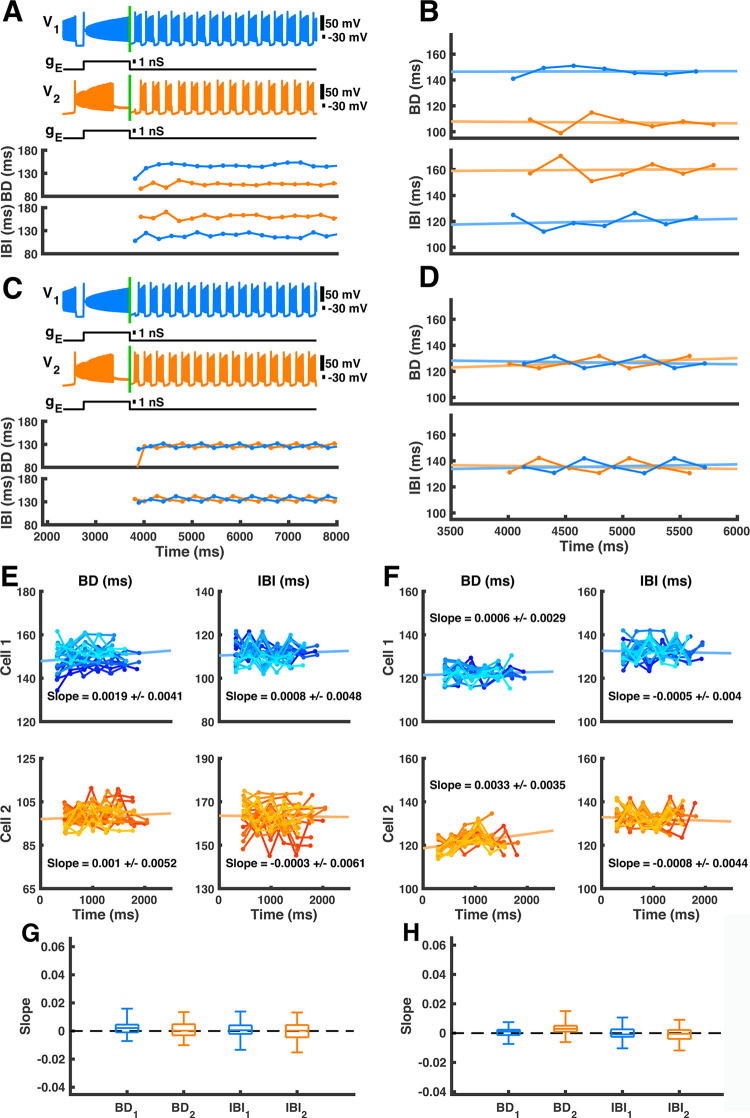
Constant *h*_*CaS*_ during transient paw-shake-like activity. **(A)** *h*_*CaS*_ was set constant in both neurons at pulse offset; this moment is marked by a vertical green line. The same example of transient activity that was used in Figs 1, 3, and 6 is shown. *h*_*CaS1*_ and *h*_*CaS2*_ were held constant at the values they had reached at that moment (*h*_*CaS1*_ = 0.0311, *h*_*CaS2*_ = 0.0242). **(B)** BD and IBI are shown for the first few bursts of trace in **A**. These burst characteristics can be compared to Fig 3B or Fig 6B. **(C)** *h*_*CaS*_ was again set constant at pulse offset (vertical green line), but in this case *h*_*CaS1*_ and *h*_*CaS2*_ were both set to the average of the values they reached at the end of the pulse (*h*_*CaS1*_ = *h*_*CaS2*_ = 0.0277). **(D)** The progression of BD and IBI of trace shown in **C**. **(E)** *h*_*CaS*_ was set constant asymmetrically after the pulse for all 286 transience cases used in Fig 5. Linear regression analysis was applied to the resulting progressions of BD and IBI and the mean linear curve fit is shown. **(F)** *h*_*CaS*_ was set constant symmetrically after the pulse for all 286 transient cases from Fig 5. Linear regression analysis was applied to each resulting progression of BD and IBI and the mean linear curve fit is shown. **(G)** Boxplot of the linear regression slopes for all 286 simulations where *h*_*CaS*_ was set constant to asymmetric values. **(H)** Boxplot of the linear regression slopes for all 286 simulations where *h*_*CaS*_ was set constant symmetrically.

In order to determine whether *h*_*NaS*_ plays any notable role in generating the asymmetric trends throughout paw-shake transience, we set *h*_*CaS*_ constant in both neurons at the moment the pulse ended using the same example of transient activity that was used in Figs 1, 3, and 6 (Fig 8A). At the moment the pulse ended, *h*_*CaS1*_ and *h*_*CaS2*_ were held constant at the values they had reached, and thereby, at different asymmetric values (*h*_*CaS1*_ = 0.0311, *h*_*CaS2*_ = 0.0242) (Fig 8). We found that while the baseline values for BD and IBI were asymmetric across the two model neurons, they remained roughly constant at those values indefinitely (Fig 8B). We applied this same procedure to all of the cases of transience previously analyzed in 20% - 40% phase range and found that this result was consistent (Fig 8E and 8G). To quantify this result, we applied linear regression analysis to the first few bursts after the pulse, where the number of bursts included was constrained to the number of bursts in the original transient paw-shake-like activity of the corresponding simulation. Since the asymmetric trends in BD and IBI disappear when *h*_*CaS*_ is held constant, the kinetics of the inactivation of slow calcium current is the sole driver of these asymmetric trends in our model.

We then performed the same procedure except that we held *h*_*CaS*_ constant at the same value for both neurons, namely at the average of the two values reached at the moment of pulse offset (Fig 8C, 8D, 8F, and 8H). We found no asymmetric trends of BD and IBI, and we found that the asymmetry in the baseline values for BD and IBI disappeared; BD and IBI both remained roughly constant at the same value for both neurons (Fig 8D, 8F, and 8H). Therefore, the kinetics of the inactivation of slow calcium current is the sole driver of asymmetry in the baseline values of BD and IBI during transient paw-shake-like activity.

### Temporal dynamics of EMG activity bursts during experimental paw-shake response

**Fig 9:**
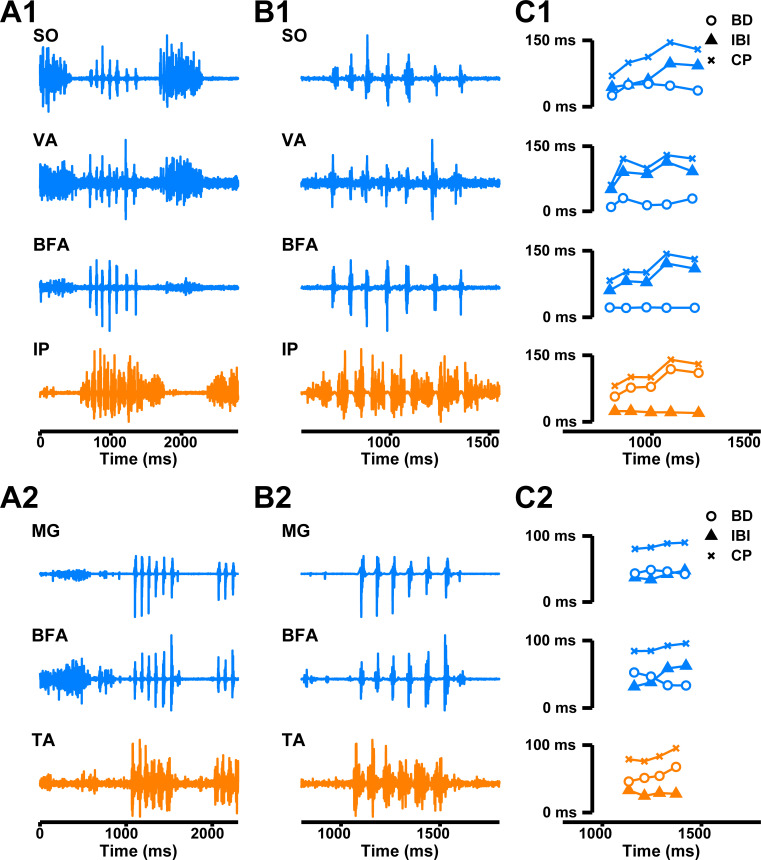
Examples of EMG extensor and flexor activity of hindlimb muscles in a paw-shake episode during locomotion. **Data of Cats J and F (Table 3).** Paw-shake EMG activity during locomotion is shown for muscles with extensor EMG bursts (*medial gastrocnemius*, MG; *soleus*, SO; *vastus lateralis*, VA; and *biceps femoris anterior*, BFA) and flexor EMG bursts (*iliopsoas*, IP and *tibialis anterior*, TA). **(A1)** EMG activity of muscles SO, VA, BFA and IP of cat J during walking and a paw-shake response. **(A2)** EMG activity of muscles MG, SO, and TA of cat F during walking and a paw-shake response. **(B1, B2)** Zoomed-in EMG traces of the paw-shake episodes in **A1** and **A2**. **(C1, C2)** Burst duration (BD), interburst interval (IBI), and cycle period (CP) for each cycle of the paw-shake responses and each muscle as a function of time. The activities of extensors and flexors are depicted in blue and orange, respectively.

**Fig 10:**
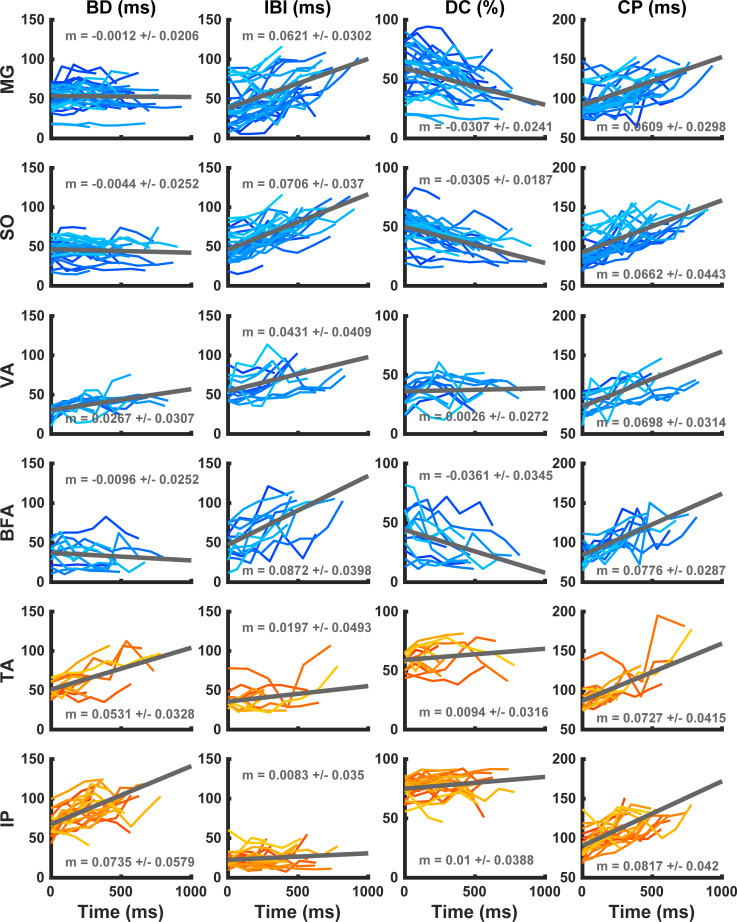
EMG burst duration (BD), interburst interval (IBI), duty cycle (DC), and cycle period (CP) of hindlimb extensor and flexor activity as functions of time during paw-shake response. Gray straight lines represent linear regression lines. Slope values shown in each panel represent the mean ± SD of the regression slope coefficient. EMG characteristics of extensor muscles (*gastrocnemius medial*, MG; *soleus*, SO; *vastus lateralis*, VA; and *biceps femoris anterior*, BFA) are indicated by blue lines; characteristics of flexor muscles (*iliopsoas*, IP and *tibialis anterior*, TA) are in orange. Data are from all 12 cats and 113 paw-shake episodes (Table 3).

During actual paw-shaking in cats, the recorded muscles demonstrated reciprocal extensor and flexor EMG activity (Fig 9A and 9B). The paw-shake CP progressively increased at each consecutive cycle within an episode (Figs 9C and 10), consistent with a previous study [19]. Since CP grows throughout a paw-shake response, one might expect that BD and IBI would both grow throughout a paw-shake response also. That was not the case for most of the muscles we recorded. BDs of flexors (TA and IP) tended to grow throughout a response, while flexor IBIs did not; on the other hand, IBIs of extensors (SO, MG, VA, and BFA) tended to grow throughout a response, while extensor BDs did not, except for VA (Figs 9C and 10). This unexpected asymmetry may offer some insight into the neural dynamics that could produce this rhythmic response. Interestingly, we also found that flexor EMG activity had a greater DC than extensor activity during paw-shaking, even though the extensor EMG activity had a greater DC during walking (Figs 9A and 9B and 10).

**Fig 11:**
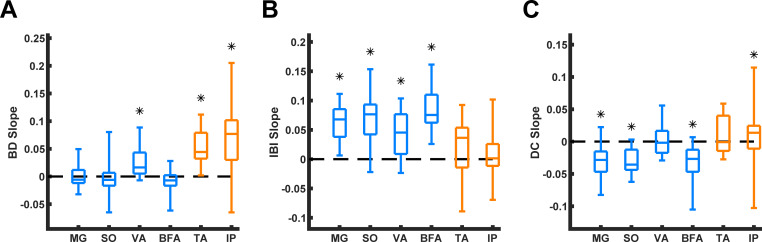
Median and quartile slopes of the linear regression lines relating EMG burst duration (BD), interbust interval (IBI), and duty cycle (DC) with time during paw shaking. Box plots depict the median slopes and quartiles of BD versus burst time **(A)**, IBI versus burst time **(B)**, and DC versus burst time **(C)** for all of the muscles. The dashed lines represent a slope of zero. Black asterisks indicate whether the linear regression slopes are statistically different from zero, with a p-value < 0.05 (the Wilcoxon signed rank test).

We calculated Pearson’s correlation coefficient (R) and performed linear regression analysis of BD, IBI, and DC for each paw-shake response within each muscle group. We used the one-sample Wilcoxon signed rank test to examine if the median linear regression slopes of BD, IBI and DC (Fig 10) were significantly different from zero. We found that for each of the hindlimb extensors (MG, SO, VA and BFA), the median IBI slopes were significantly positive (Figs 10 and 11B; for MG, p = 3.8x10^-6^, n = 28 paw-shake episodes; for SO, p = 4.7x10^-6^, n = 28; for VA, p = 6.8x10^-3^, n = 12; for BFA, p = 4.9x10^-4^, n = 12). The median BD slopes for these muscles, on the other hand, were not different from zero, except for VA (Figs 10 and 11A; for MG, p = 0.399, n = 28; for SO, p = 0.151, n = 28; for BFA, p = 0.233, n = 12; while for VA p = 4.9x10^-3^, n = 12). The DC slopes of the extensor bursts were significantly less than zero (except for VA), i.e. DC of these bursts decreased with consecutive cycles of paw-shaking (Figs 10 and 11C; for MG, p = 1.5x10^-5^, n = 28; for SO, p = 5.3x10^-6^, n = 28; for BFA, p = 9.8x10^-4^, n = 12); but the VA DC was not different from zero (p = 1.0, n = 12).

After using the same Wilcoxon signed rank test on slopes of EMG bursts of flexor muscles IP and TA (Figs 9 and 10), we obtained mostly opposite results. Specifically, the median BD slopes were significantly positive for these muscles (Figs 10 and 11A; for IP, p = 2.1x10^-4^, n = 21 and for TA, p = 4.9x10^-4^, n = 12), while the IBI slopes were not significantly different from zero for IP and TA (Figs 10 and 11B; for IP, p = 0.357, n = 21; and for TA, p = 0.151, n = 12).

The DC slope for IP was significantly greater than zero (p = 0.0496, n = 21). The median DC slope for TA was positive but not significantly different from zero (p = 0.677, n = 12).

## Discussion

In this study, we demonstrated that the model of a multistable half-center oscillator CPG, capable of producing steady-state slow locomotor-like and fast paw-shake-like rhythms [17,18], could also produce a fast paw-shake-like rhythm as a transient response to a perturbation of the slow steady-state locomotor-like rhythm (Fig 1). The transient activity was asymmetric with different evolution rates for BD, IBI, and DC of the two half-centers throughout the transient response, despite that the two model half-centers are entirely identical (Figs 3–5). In the model, we determined that this asymmetry resulted from the anti-phasic activity occurring at the time identical current pulses were applied to both neurons. More specifically, the asymmetry resulted from the different levels of inactivation of the slow calcium currents between the two neurons at the time of pulse offset and the different progressions of these levels throughout the transient activity (Figs 6 and 7).

We also recorded EMG activity of selected hindlimb muscles while cats performed paw-shaking during walking. Measured paw-shake cycle periods increased in consecutive paw-shake cycles (Figs 9 and 10) in accordance with previous studies [19]. We demonstrated for the first time that EMG burst duration and interburst interval evolve asymmetrically in consecutive paw-shake cycles of hindlimb muscles with flexor- and extensor-related activity (Figs 9–11). This asymmetry was similar to the transient dynamics of the model CPG half-centers (Figs 3 and 5).

In the next sections, we discuss these findings in the light of the potential role of multistable CPGs and their possible operation during locomotion and paw-shaking in the cat.

### Functional transient activity in biological systems

Central pattern generators are often modeled to produce stable steady-state rhythmic patterns, and each such pattern is an attractor state of the system. When a system exhibiting a steady-state pattern, e.g. a locomotor pattern, is perturbed, some transient activity may occur before the system returns to that attractor state and resumes its steady-state rhythmic pattern [14,15,28]. This transient activity is often not considered in modeling studies, although it may represent functional activity of the network with properties necessary for producing an appropriate response to a stimulus [14,15,28–30].

Studies have shown in various neural circuits of different organisms that transient activity can have functional purposes sometimes more so than steady state activity. For example, olfactory responses are encoded by transient dynamics in many investigated species [14,15,31,32]. In locusts, when an odor is presented in a short puff of air, transient oscillations of membrane potential occur in specific neurons of the antennal lobe depending on the odor presented [31]. Each neuron responds specifically to a small set of odors, and the transient pattern of the evoked oscillation is specific to the odor [31]. If the stimulus is present for more than 2–3 seconds, then the oscillations reach a stable attractor state, and odor specificity decreases during this stable activity [14,31]. The above example demonstrates that the properties of the transient responses to short stimuli may be more important for functionality than the steady-state pattern in locust olfaction. Transient dynamics are known to produce reliable responses to specific stimuli in other systems as well, such as mammalian learning and memory processes and cortical visual processing [15,16,33].

We propose that a mechanism similar to the one we have modeled here could apply to other species where a brief behavior is required to respond to some environmental stimulus. For instance, the struggling behavior in Xenopus tadpoles and larval zebrafish [6,11,12,34] and the scratching behavior in turtles [5,6,35,36]. Struggling and scratching are transient, reflexive behaviors that could be generated by a transient response of the swimming CPG in either organism.

### Potential transient dynamics of locomotor CPG controlling the paw-shake-like pattern

Given that the recorded paw-shake cycle periods change in each successive cycle (Figs 9–11 and [19]), we propose that cat paw-shaking may result from a locomotor CPG’s transient activity. Paw-shaking can be elicited in deafferented spinalized cats [37] and fictive paw-shake-like responses can be produced in spinal-transected curarized cat preparations [38], suggesting that the paw-shaking rhythm is generated by a spinal CPG. Although motion-dependent somatosensory feedback affects the centrally generated paw-shake activity patterns, these effects appear to be limited to two muscle groups responsible for the atypical muscle synergy during paw-shaking–the *vasti* (VA) and *tibialis anterior* (TA) muscles [37].

In the current study, we investigated the question of whether paw-shake responses can be produced as a transient response of the multifunctional locomotor CPG. Our HCO CPG model can exhibit transient paw-shake-like activity in two different types of parameter regimes, (1) when the model exhibits coexistence of paw-shake-like activity and locomotor-like activity, and (2) when the model exhibits only locomotor-like activity but is set nearby the stable paw-shake-like regime in parameter space. In (2), having CPG parameters set in the vicinity of the range supporting the fast paw-shake-like regime as an attractor relates to the concept of a conditional burster: a neuron producing endogenous bursting activity under certain physiological conditions, for example under certain neuromodulatory tone. If both slow and fast regimes are attractors as in case (1) then transient paw-shake-like activity could be induced due to a slow passage near basin of attraction of the fast regime as a result of perturbation which shifted trajectory of HCO activity close to the border between basins of attraction of the two regimes but did not make it cross the border. Our model uses a relatively novel mechanism for multistability of bursting regimes with largely distinct periods that involves the different kinetics of inactivation of two intrinsic slow currents [17]. These two slow currents were built with largely distinct time constants of inactivation and different voltages of half-inactivation in order to produce two rhythmic bursting regimes with cycle periods that differ by an order of magnitude, the locomotor regime (1 Hz) and the paw-shake-like regime (10 Hz). The model was built so that the kinetics of inactivation of the slow Ca^++^ (*I*_*CaS*_) and Na^+^ (*I*_*NaS*_) currents would drive the kinetics of inactivation of the slow and fast regime, respectively. When transient activity is generated in our model, slow Na^+^ current is still the main driving current of the transient fast activity, but slow Ca^++^ current controls the way burst duration and interburst interval change over time and the way the transient activity terminates and returns to the slow rhythm. The transient rhythmic bursting is reliably controlled by the slowest variable of the system, *h*_*CaS*_, which drives the slow bursting rhythm, locomotor-like bursting.

While it has been shown in other CPG models that asymmetric external input can create asymmetric burst characteristics in a symmetric model [39], we show that entirely symmetric external input can create asymmetric burst characteristics in a symmetric model, due to the inherent asymmetry involved in anti-phasic activity. Using a pulse applied symmetrically to both neurons, we could obtain an asymmetric response characterized by an asymmetric evolution of BD and IBI for neurons #1 and #2, depending on the phase of pulse onset. This asymmetry occurred due to the anti-phasic activity of the two neurons. The same pulse was applied to both neurons, but if it was applied during the silent phase of neuron #1 and the spiking phase of neuron #2, then BD in the elicited paw-shake-like response would grow faster for neuron #2 and IBI would grow faster for neuron #1. Since neuron #2 was spiking, the slow calcium current was inactivated, i.e. its inactivation state variable *h*_*CaS2*_ was decaying towards 0. At the same time, the membrane potential of neuron #1 was hyperpolarized, and the slow calcium current was deinactivated, its inactivation state variable *h*_*CaS1*_ was growing towards 1. Thus, *h*_*CaS1*_ was larger than *h*_*CaS2*_ at the time the pulse was applied due to the asymmetric removal of inactivation of the slowest inward current. During the pulse, the slow calcium current inactivated in both neurons, i.e. the values of *h*_*CaS*_ in both neurons were decreasing, but at the end of the pulse, *h*_*CaS*_ was still greater for neuron #1 than for neuron #2 (Fig 6D). During transient activity for neuron #2, the value of *h*_*CaS*_ at the beginning of the burst increased at each consecutive burst causing BD to increase throughout the transient response, and this, in turn, caused IBI of neuron #1 to increase throughout the transient response (Fig 6). During transient activity for neuron #1, the values of *h*_*CaS*_ at the beginning of the burst made a very slight U-shape as it progressed, causing BD_1_ of neuron to remain roughly constant throughout transience, which caused IBI_2_ to also remain roughly constant (Fig 6). Therefore, although the current *I*_*NaS*_ drives the stable fast rhythm, the current *I*_*CaS*_, driving the stable slow rhythm, controls the duration and dynamics of the transient fast activity. During the stable fast rhythm, *I*_*CaS*_ is completely inactivated so when *I*_*CaS*_ is only slightly deinactivated, it has a large effect on the BDs of the two neurons (Fig 5). In our model, the value of *h*_*CaS*_ only increased to around 0.02 before the transition to the slow rhythm (Fig 7). Interestingly, these small differences in the *h*_*CaS*_ values between neurons #1 and #2 were able to create substantial asymmetry in burst durations between the two neurons (Figs 6 and 7).

It has been theorized that slowly inactivating sodium current is the main driving current of the mammalian locomotor rhythm [20]. There is experimental evidence indicating that slowly inactivating sodium current plays an important role in the generation of fictive locomotion in the isolated rodent spinal cord [40]. Although slowly inactivating low-voltage-activated calcium current is the main driving current of the locomotor-like rhythm in our model, slowly inactivating sodium current is still required to generate locomotor-like activity. There is also experimental evidence indicating that slowly inactivating low-voltage-activated calcium current contributes to the fictive locomotor rhythm in the isolated neonatal mouse spinal cord, but it has not been determined whether this current is crucial to the generation of this rhythm [41,42].

### Possible organization of a mammalian multifunctional locomotor CPG capable of producing faster transient rhythmic behaviors

In this study, we investigated a simple half-center oscillator CPG generating two phases (flexor and extensor) of a steady-state rhythmic locomotor-like behavior and a paw-shake-like transient activity. One limitation of our model is that it does not consider sensory feedback despite that motion-dependent somatosensory feedback affects the centrally generated experimental paw-shake activity patterns. However, these effects appear to be limited to two muscle groups responsible for the atypical muscle synergy during paw-shaking–the *vasti* (VA) and *tibialis anterior* (TA) muscles [37]. The phase of the VA activity burst in the cycle of real and fictive locomotion [25,43–45] or real and fictive paw-shaking [19,37,38,46,47] is different from activity burst phases of other pure hindlimb extensors. This suggests a unique organization of spinal networks controlling VA motoneurons. In this study, we found that VA did not have the same BD trends as other extensors, which is consistent with the previous findings that VA’s phase relationships during paw-shaking are also different from other extensors [19,25,37,38,46,47].

We have previously shown by using a neuromechanical model of cat hindlimbs that a locomotor CPG comprising a half-center oscillator rhythm generator and a basic reflex network is capable of reproducing both walking and paw-shaking [17]. In that extended model, the flexor and extensor half-centers activated flexor and extensor motoneurons, respectively, and the neuromechanical model reproduced co-activation of knee extensor *vasti* and ankle flexor *tibialis anterior*, a.k.a. the paw-shake mixed synergy [19], due to additional strong excitatory input from spindle length-sensitive afferents of *vasti* being stretched during the flexor phase. Thus, our previous modeling results, the reciprocal activity of hindlimb muscles with flexor- and extensor-related EMG bursts in this (Fig 9) and other studies [17], as well as experimental studies of fictive paw-shake-like activity patterns generated without motion-dependent sensory input in the cat [38], have demonstrated that most of flexor and extensor hindlimb motoneuronal pools have reciprocal activity patterns consistent with receiving flexor and extensor inputs from a half-center oscillator CPG. There have been suggestions that the same or a largely shared CPG rhythm generator with flexor and extensor half-centers could generate both slow locomotor-like and fast scratch-like fictive activity patterns in the cat [48,49]. These suggestions were based on similar effects of deletions and afferent stimulations seen during fictive locomotion and scratching [48,49]. Although the duration of flexor and extensor phases are different between fictive locomotion and fictive scratching, suggesting that these two behaviors could be controlled by two specialized spinal rhythm-generating mechanisms [50], these observations appear consistent with the behavior of a single multistable HCO CPG during locomotor-like and transient paw-shake like activities (Figs 5, 6 and 9–11).

Although the exact structure of the mammalian spinal locomotor CPG is still debated, all current CPG schematics contain HCO CPG elements that provide excitatory inputs to flexor and extensor motoneurons of all muscles throughout the hindlimb [51,52]. While the more realistic model of the HCO locomotion CPG in mammals would consist of two inhibitory populations of neurons carrying mutual inhibition between two excitatory populations of neurons, we model this HCO with a single neuron representing an excitatory neural population with synaptic transmission representing signals carried by an inhibitory population. This simplification is a common practice used in computational modeling studies assuming highly synchronized activity of the two populations making up one half-center [36,53]. This simplification would miss patterns of activity with more complex relationships between neurons within and between excitatory and inhibitory populations. We suggest that our simple model represents the key temporal features of the measured EMG signals of different cat’s muscles during locomotor and paw shaking rhythmic movements [36,53]. Our simplified HCO model may provide useful insights into potential mechanisms of the transition from locomotor-like rhythms to faster paw-shake like activities and their generation.

In summary, we investigated a model of a multifunctional locomotor CPG, which exhibited coexistence of a slow locomotor regime and a fast paw-shaking regime and found that transient paw-shake-like responses exhibit functionally asymmetric phase duration changes between the half-centers, and that this asymmetry depends on the phase of the locomotor-like rhythm at which the perturbation was applied. In the model, the asymmetry is caused by asymmetric levels of inactivation across the two half-centers of the slowly inactivating inward current that drives the locomotor-like rhythm. In experiments, muscle activity driving a paw-shake response evoked during cat locomotion is transient in nature and consists of high-frequency bursts with increasing cycle durations and a similar asymmetry in phase duration changes. Our study makes a general prediction that multifunctionality of a CPG producing two patterns, one with a slow steady rhythm and the other with a fast transient rhythm, could potentially be identified by trends exhibited during the fast rhythm. The fast rhythm would exhibit asymmetry in its pattern imposed by the state of the shared slow variable participating in the slow rhythm.

## Supporting information

S1 Data Folder contains 44 supplementary data files each corresponding to a single episode of paw shaking. File name of each file has the following structure: SupplementaryFileNumber_Data_SessionNumber_AnimalCode_EpisodeNumber.txt. Example: S1_Data_7_3-25-0_bo_9F.txt, where S1 is the supplementary data file number 7, 3-25-0 is session number, bo is animal code, 9F is episode number, and txt is file type extension. Each column of each file contains raw EMG signal of a specific muscle in mV sampled at 3000 Hz and band-pass filtered (Butterworth zero-lag filter, 30 Hz– 1000 Hz, 3 dB). First line of each file contains muscle name abbreviations (see Table 3 in the manuscript): MG, medial gastrocnemius; SO, soleus; BFS, biceps femoris anterior; IP, iliopsoas; TA, tibialis anterior; VA, vastus lateralis and vastus medialis.
(ZIP)

## References

[1] Berkowitz A. Expanding our horizons: central pattern generation in the context of complex activity sequences. J Exp Biol. 2019;222(Pt 20). doi: 10.1242/jeb.192054 31615858

[2] Grillner S. Biological pattern generation: the cellular and computational logic of networks in motion. Neuron. 2006;52(5):751–66. doi: 10.1016/j.neuron.2006.11.008 17145498

[3] McCrea DA, Rybak IA. Organization of mammalian locomotor rhythm and pattern generation. Brain Res Rev. 2008;57(1):134–46. doi: 10.1016/j.brainresrev.2007.08.006 17936363 PMC2214837

[4] Marder E, Calabrese RL. Principles of rhythmic motor pattern generation. Physiol Rev. 1996;76(3):687–717. doi: 10.1152/physrev.1996.76.3.687 8757786

[5] Berkowitz A. Both shared and specialized spinal circuitry for scratching and swimming in turtles. Journal of Comparative Physiology A. 2002;188(3):225–34. doi: 10.1007/s00359-002-0297-7 11976891

[6] Berkowitz A, Roberts A, Soffe SR. Roles for multifunctional and specialized spinal interneurons during motor pattern generation in tadpoles, zebrafish larvae, and turtles. Frontiers in behavioral neuroscience. 2010;4:36. doi: 10.3389/fnbeh.2010.00036 20631847 PMC2903196

[7] Briggman KL, Kristan WB. Imaging dedicated and multifunctional neural circuits generating distinct behaviors. Journal of Neuroscience. 2006;26(42):10925–33. doi: 10.1523/JNEUROSCI.3265-06.2006 17050731 PMC6674766

[8] Cangiano L, Grillner S. Fast and Slow Locomotor Burst Generation in the Hemispinal Cord of the Lamprey. Journal of Neurophysiology. 2003;89(6):2931–42. doi: 10.1152/jn.01100.2002 12611971

[9] Cangiano L, Hill R, Grillner S. The hemisegmental locomotor network revisited. Neuroscience. 2012;210:33–7. doi: 10.1016/j.neuroscience.2012.03.007 22433298

[10] Jing J, Weiss KR. Neural Mechanisms of Motor Program Switching inAplysia. Journal of Neuroscience. 2001;21(18):7349–62. doi: 10.1523/JNEUROSCI.21-18-07349.2001 11549745 PMC6762995

[11] Liao JC, Fetcho JR. Shared versus specialized glycinergic spinal interneurons in axial motor circuits of larval zebrafish. Journal of Neuroscience. 2008;28(48):12982–92. doi: 10.1523/JNEUROSCI.3330-08.2008 19036991 PMC2677998

[12] Soffe S. Two distinct rhythmic motor patterns are driven by common premotor and motor neurons in a simple vertebrate spinal cord. Journal of Neuroscience. 1993;13(10):4456–69. doi: 10.1523/JNEUROSCI.13-10-04456.1993 8410198 PMC6576385

[13] Trejo A, Tapia J, De la Torre Valdovinos B, Huidobro N, Flores G, Flores-Hernandez J, et al. Transition of pattern generation: the phenomenon of post-scratching locomotion. Neuroscience. 2015;288:156–66. doi: 10.1016/j.neuroscience.2014.12.038 25556832

[14] Mazor O, Laurent G. Transient dynamics versus fixed points in odor representations by locust antennal lobe projection neurons. Neuron. 2005;48(4):661–73. doi: 10.1016/j.neuron.2005.09.032 16301181

[15] Rabinovich M, Huerta R, Laurent G. Neuroscience. Transient dynamics for neural processing. Science. 2008;321(5885):48–50. doi: 10.1126/science.1155564 18599763

[16] Jones LM, Fontanini A, Sadacca BF, Miller P, Katz DB. Natural stimuli evoke dynamic sequences of states in sensory cortical ensembles. Proc Natl Acad Sci U S A. 2007;104(47):18772–7. doi: 10.1073/pnas.0705546104 18000059 PMC2141852

[17] Bondy B, Klishko AN, Edwards DH, Prilutsky BI, Cymbalyuk G. Control of cat walking and paw-shake by a multifunctional central pattern generator. In: Prilutsky BI, Edwards DH, editors. Neuromechanical Modeling of Posture and Locomotion. New York, NY: Springer; 2016. p. 333–59.

[18] Parker J, Bondy B, Prilutsky BI, Cymbalyuk G. Control of transitions between locomotor-like and paw shake-like rhythms in a model of a multistable central pattern generator. Journal Neurophysiol. 2018;120(3):1074–89. 10.1152/jn.00696.2017 PMC6171058 29766765

[19] Smith JL, Hoy MG, Koshland GF, Phillips DM, Zernicke RF. Intralimb coordination of the paw-shake response: a novel mixed synergy. J Neurophysiol. 1985;54(5):1271–81. doi: 10.1152/jn.1985.54.5.1271 4078616

[20] Rybak IA, Shevtsova NA, Lafreniere-Roula M, McCrea DA. Modelling spinal circuitry involved in locomotor pattern generation: insights from deletions during fictive locomotion. The Journal of physiology. 2006;577(2):617–39. doi: 10.1113/jphysiol.2006.118703 17008376 PMC1890439

[21] Shevtsova NA, Rybak IA. Organization of flexor–extensor interactions in the mammalian spinal cord: insights from computational modelling. The Journal of physiology. 2016;594(21):6117–31. doi: 10.1113/JP272437 27292055 PMC5088238

[22] Hodgkin AL, Huxley AF. A quantitative description of membrane current and its application to conduction and excitation in nerve. J Physiol. 1952;117(4):500–44. doi: 10.1113/jphysiol.1952.sp004764 12991237 PMC1392413

[23] Mehta R, Prilutsky BI. Task-dependent inhibition of slow-twitch soleus and excitation of fast-twitch gastrocnemius do not require high movement speed and velocity-dependent sensory feedback. Frontiers in physiology. 2014;5:410. doi: 10.3389/fphys.2014.00410 25389407 PMC4211390

[24] Prilutsky BI, Maas H, Bulgakova M, Hodson-Tole EF, Gregor RJ. Short-term motor compensations to denervation of feline soleus and lateral gastrocnemius result in preservation of ankle mechanical output during locomotion. Cells, tissues, organs. 2011;193(5):310–24. doi: 10.1159/000323678 21411965 PMC3128141

[25] Markin SN, Lemay MA, Prilutsky BI, Rybak IA. Motoneuronal and muscle synergies involved in cat hindlimb control during fictive and real locomotion: a comparison study. J Neurophysiol. 2012;107(8):2057–71. doi: 10.1152/jn.00865.2011 22190626 PMC3331602

[26] Maas H, Gregor RJ, Hodson-Tole EF, Farrell BJ, English AW, Prilutsky BI. Locomotor changes in length and EMG activity of feline medial gastrocnemius muscle following paralysis of two synergists. Exp Brain Res. 2010;203(4):681–92. doi: 10.1007/s00221-010-2279-2 20458472 PMC2880237

[27] Hodson-Tole EF, Pantall AL, Maas H, Farrell BJ, Gregor RJ, Prilutsky BI. Task dependent activity of motor unit populations in feline ankle extensor muscles. J Exp Biol. 2012;215:3711–22. doi: 10.1242/jeb.068601 22811250 PMC3470066

[28] Rabinovich M, Volkovskii A, Lecanda P, Huerta R, Abarbanel H, Laurent G. Dynamical encoding by networks of competing neuron groups: winnerless competition. Physical review letters. 2001;87(6):068102. doi: 10.1103/PhysRevLett.87.068102 11497865

[29] Bazhenov M, Stopfer M, Rabinovich M, Huerta R, Abarbanel HD, Sejnowski TJ, et al. Model of transient oscillatory synchronization in the locust antennal lobe. Neuron. 2001;30(2):553–67. doi: 10.1016/s0896-6273(01)00284-7 11395014 PMC2900257

[30] Bazhenov M, Stopfer M, Rabinovich M, Abarbanel HD, Sejnowski TJ, Laurent G. Model of cellular and network mechanisms for odor-evoked temporal patterning in the locust antennal lobe. Neuron. 2001;30(2):569–81. doi: 10.1016/s0896-6273(01)00286-0 11395015 PMC2907737

[31] Laurent G, Davidowitz H. Encoding of olfactory information with oscillating neural assemblies. Science. 1994;265(5180):1872–5. doi: 10.1126/science.265.5180.1872 17797226

[32] Stopfer M, Jayaraman V, Laurent G. Intensity versus identity coding in an olfactory system. Neuron. 2003;39(6):991–1004. doi: 10.1016/j.neuron.2003.08.011 12971898

[33] Lin L, Osan R, Shoham S, Jin W, Zuo W, Tsien JZ. Identification of network-level coding units for real-time representation of episodic experiences in the hippocampus. Proc Natl Acad Sci U S A. 2005;102(17):6125–30. doi: 10.1073/pnas.0408233102 15833817 PMC1087910

[34] Soffe S. The pattern of sensory discharge can determine the motor response in young Xenopus tadpoles. Journal of Comparative Physiology A. 1997;180(6):711–5. doi: 10.1007/s003590050085 9190047

[35] Berkowitz A. Multifunctional and specialized spinal interneurons for turtle limb movements. Annals of the New York Academy of Sciences. 2010;1198(1):119–32. doi: 10.1111/j.1749-6632.2009.05428.x 20536926

[36] Hao Z-Z, Spardy LE, Nguyen EB, Rubin JE, Berkowitz A. Strong interactions between spinal cord networks for locomotion and scratching. Journal of neurophysiology. 2011;106(4):1766–81. doi: 10.1152/jn.00460.2011 21734103

[37] Koshland GF, Smith JL. Mutable and immutable features of paw-shake responses after hindlimb deafferentation in the cat. J Neurophysiol. 1989;62(1):162–73. doi: 10.1152/jn.1989.62.1.162 2754470

[38] Pearson KG, Rossignol S. Fictive motor patterns in chronic spinal cats. J Neurophysiol. 1991;66(6):1874–87. doi: 10.1152/jn.1991.66.6.1874 1812222

[39] Daun S, Rubin JE, Rybak IA. Control of oscillation periods and phase durations in half-center central pattern generators: a comparative mechanistic analysis. Journal of computational neuroscience. 2009;27(1):3–36. doi: 10.1007/s10827-008-0124-4 19130197 PMC2844522

[40] Ausborn J, Snyder AC, Shevtsova NA, Rybak IA, Rubin JE. State-dependent rhythmogenesis and frequency control in a half-center locomotor CPG. J Neurophysiol. 2018;119(1):96–117. doi: 10.1152/jn.00550.2017 28978767 PMC5866471

[41] Anderson TM, Abbinanti MD, Peck JH, Gilmour M, Brownstone RM, Masino MA. Low-threshold calcium currents contribute to locomotor-like activity in neonatal mice. J Neurophysiol. 2012;107(1):103–13. doi: 10.1152/jn.00583.2011 21994264 PMC3349703

[42] Dougherty KJ, Ha NT. The rhythm section: an update on spinal interneurons setting the beat for mammalian locomotion. Current opinion in physiology. 2019;8:84–93. doi: 10.1016/j.cophys.2019.01.004 31179403 PMC6550992

[43] Higgin D, Krupka A, Maghsoudi OH, Klishko AN, Nichols TR, Lyle MA, et al. Adaptation to slope in locomotor-trained spinal cats with intact and self-reinnervated lateral gastrocnemius and soleus muscles. J Neurophysiol. 2020;123(1):70–89. doi: 10.1152/jn.00018.2019 31693435 PMC6985865

[44] Krouchev N, Kalaska JF, Drew T. Sequential activation of muscle synergies during locomotion in the intact cat as revealed by cluster analysis and direct decomposition. J Neurophysiol. 2006;96(4):1991–2010. doi: 10.1152/jn.00241.2006 16823029

[45] Klishko AN, Akyildiz A, Mehta-Desai R, Prilutsky BI. Common and distinct muscle synergies during level and slope locomotion in the cat. J Neurophysiol. 2021;126(2):493–515. doi: 10.1152/jn.00310.2020 34191619 PMC8409955

[46] Barbeau H, Julien C, Rossignol S. The effects of clonidine and yohimbine on locomotion and cutaneous reflexes in the adult chronic spinal cat. Brain research. 1987;437(1):83–96. doi: 10.1016/0006-8993(87)91529-0 3427484

[47] Barbeau H, Rossignol S. The effects of serotonergic drugs on the locomotor pattern and on cutaneous reflexes of the adult chronic spinal cat. Brain research. 1990;514(1):55–67. doi: 10.1016/0006-8993(90)90435-e 2357531

[48] Lafreniere-Roula M, McCrea DA. Deletions of rhythmic motoneuron activity during fictive locomotion and scratch provide clues to the organization of the mammalian central pattern generator. J Neurophysiol. 2005;94(2):1120–32. doi: 10.1152/jn.00216.2005 15872066

[49] Stecina K, Quevedo J, McCrea DA. Parallel reflex pathways from flexor muscle afferents evoking resetting and flexion enhancement during fictive locomotion and scratch in the cat. J Physiol. 2005;569(Pt 1):275–90. doi: 10.1113/jphysiol.2005.095505 16141269 PMC1464219

[50] Frigon A, Gossard JP. Evidence for specialized rhythm-generating mechanisms in the adult mammalian spinal cord. The Journal of neuroscience: the official journal of the Society for Neuroscience. 2010;30(20):7061–71. 10.1523/JNEUROSCI.0450-10.2010 PMC6632641 20484648

[51] Grillner S, Kozlov A. The CPGs for limbed locomotion-facts and fiction. Int J Mol Sci. 2021;22(11). doi: 10.3390/ijms22115882 34070932 PMC8198624

[52] Rybak IA, Dougherty KJ, Shevtsova NA. Organization of the mammalian locomotor CPG: review of computational model and circuit architectures based on genetically identified spinal interneurons(1,2,3). eNeuro. 2015;2(5). doi: 10.1523/ENEURO.0069-15.2015 26478909 PMC4603253

[53] Snyder AC, Rubin JE. Conditions for multi-functionality in a rhythm generating network inspired by turtle scratching. The Journal of Mathematical Neuroscience (JMN). 2015;5(1):1–34. 10.1186/s13408-015-0026-5 PMC4504876 26185063

